# LYRASIS Learning

**DOI:** 10.5195/jmla.2021.1343

**Published:** 2021-10-01

**Authors:** Karen L. Yacobucci

**Affiliations:** 1 karen.yacobucci@nyulangone.org, NYU Health Sciences Library, New York, NY

## Abstract

**LYRASIS Learning.** LYRASIS, 1438 West Peachtree Street, NW, Suite 150, Atlanta, GA 30309; 800.999.8558; https://www.lyrasis.org/Leadership/Pages/LYRASIS-Learning.aspx; individual class pricing available for members and nonmembers, contact for member pricing.

## OVERVIEW

Created in 2009, LYRASIS is a nonprofit membership organization based in the United States whose mission “is to support enduring access to the world's shared academic, scientific and cultural heritage through leadership in open technologies, content services, digital solutions and collaboration with archives, libraries, museums and knowledge communities worldwide” [1].

LYRASIS Learning is a professional development and continuing education platform that provides access to online classes and training. LYRASIS credits the creation of LYRASIS Learning from member feedback, citing the “need for high quality training at scale, allowing members to upskill multiple staff simultaneously without cost-prohibitive registration fees, and simplified billing” [2].

## DESCRIPTION

The LYRASIS Learning platform largely supports access to learning content designed for employees of libraries, archives, museums, and cultural heritage institutions. Classes cover a variety of subject matter and include topics in technology, preservation, digitization, leadership, management, and topics relevant for cultural heritage professionals.

LYRASIS Learning provides access to live, online classes as well as unlimited access to the accompanying Learning Library, which includes an archive of over 250 prerecorded classes available on-demand. New content is added monthly and becomes available in the Learning Library shortly after live sessions take place [3].

Live sessions and classes are available for registration to both LYRASIS members and nonmembers. Members are eligible for a discounted class price. For an additional fee, LYRASIS members can subscribe to LYRASIS Learning, providing the added advantage of unlimited free access to live classes and all content housed within the Learning Library.

Live classes are published regularly on LYRASIS's website and through their online course catalog, which includes class dates and times, class names, instructor name(s) that hyperlink to their biographical information, LYRASIS member and nonmember pricing, as well as a link to register.

Upon registering, an automated email is sent confirming registration. A separate email is sent with login information for the registered sessions. Captioning services for classes are available upon request by contacting LYRASIS's Educational Services via email.

Classes range in duration from one to three hours, with three-hour classes being broken up into two 1.5-hour sessions. Breaking down longer-running sessions provides the added benefit of making class content easier for attendees to digest.

Once registered, class attendees receive a follow-up email that includes course materials, the instructor's contact information, and a link to the class recording. Reminder emails are also sent to registrants thirty minutes prior to class and include information on using Adobe Connect and Zoom, the two main programs used to conduct live classes. In addition, users are eligible to receive a Certificate of Participation if they complete a LYRASIS Evaluation Form via a link included in the confirmation email.

## SIMILAR PRODUCTS

Very few online learning platforms cater to topics relevant for learners in library and information science communities. LinkedIn Learning (formerly Lynda.com) is a popular continuing education resource with over 16,000 online classes in more than seven languages, but its primary course content is focused broadly on subjects pertaining to business, creative topics, and technology, many of which are not applicable to the library and information science community [4].

One comparable learning resource would be OCLC's WebJunction. Launched by OCLC in 2003, WebJunction provides access to webinars and self-paced learning modules pertaining to library-focused content. Access to WebJunction is free, but a registered account is required. Although WebJunction has been available to learners for a historically longer period of time and currently includes a greater number of online webinars and learning modules, LYRASIS Learning appears to be adding content at a competitive pace. It is also worth noting that although both platforms are geared toward library-focused content, the platforms differ in what types of classes are available. For example, when searching WebJunction for classes related to the subject of copyright, no results were found. However, the same subject search across LYRASIS Learning content resulted in seven videos pertaining to copyright.

## CLASS INSTRUCTORS

Classes are primarily taught by information professionals from a variety of different academic institutions and professional organizations. LYRASIS Learning makes it easy to learn more about class instructors by including a link to their biographical information located in the class listings. Learners are also provided the opportunity to communicate directly with instructors at any time by emailing them through an online form.

In order to teach a class through the LYRASIS Learning platform, applicants must submit the following information using a New Instructor Interest Form [5]:

- Contact information
- Teaching topics
- A brief description of the instructor's approach to teaching
- Summary of teaching experience
- Resume or curriculum vitae
- A link to a prerecorded class or presentation, or a slide deck used for a class or presentation

Instructors can apply to develop live, online classes, and once a New Instructor Interest Form has been received, potential instructors should hear back within approximately two weeks. Compensation for instructors is provided and is based on the duration of the session.

In addition to the instructor, a LYRASIS technical support representative is present for each class. This role allows for the flow of the live classes to continue uninterrupted in the event class participants encounter any technical challenges. Since all participants are muted during the class, this support role also facilitates communication between participants with questions and the instructor.

## USABILITY

The LYRASIS Learning Library website is well designed, uncomplicated, and easy to navigate. Video content is grouped into channels by subject, making it easy to identify and locate specific video content. Selecting a channel allows users to view all videos within the channel's collection in a grid layout, as the page displays the videos with equal hierarchy. This layout makes it easy for users to visually scan the content on the channel's page. When scrolling over a video, the class date, description, running time, and number of views comes into display.

## TECHNICAL REQUIREMENTS

Online access to LYRASIS Learning is facilitated through IP registration, although online content can also be provided through a username and password login. Access to live classes is provided through Adobe Connect or Zoom, both of which are available for free download. Live classes are recorded in order to make the content available for viewing on-demand at a later date, but participant video screens and names are not shown during live classes, and therefore a participant's identity is not captured in the recording.

## QUALITY AND CUSTOMER SUPPORT

LYRASIS Learning does an excellent job of ensuring good audio and visual quality of its live classes and on-demand video content. Approximately thirty minutes prior to each class, a technical support representative conducts a sound check and review of slides with the instructor. This provides an opportunity for both the support representative and the instructor to address any technical concerns before the scheduled class, reducing time spent during class to address technical challenges participants may encounter.

LYRASIS Learning provides excellent customer support and clear communication surrounding what to expect with each live class. Their contact information is clearly noted in email communications as well as on their online website. They are also highly responsive to email and provide follow-up to inquiries in a timely manner.

## CONCLUSION

Unlike many other online learning platforms, LYRASIS Learning is unique in that its educational content is developed specifically for information professionals of all skill levels who seek high-quality training. Information professionals, particularly those working in academic environments, tend to be incentivized for continuing education and professional development growth, and LYRASIS Learning provides opportunities for both. The LYRASIS Learning online course catalog provides a wide variety of classes that are useful for the audience they purport to serve. The pricing options for live classes are flexible for both LYRASIS members and nonmembers, and pricing for LYRASIS Learning is reasonable based on the quality of content. LYRASIS Learning is a viable option for organizations and institutions seeking to upskill staff while also controlling the cost of trainings through periods of budgetary cuts and hiring freezes.
